# *Saccharomyces cerevisiae*: a nomadic yeast with no niche?

**DOI:** 10.1093/femsyr/fov009

**Published:** 2015-02-27

**Authors:** Matthew R. Goddard, Duncan Greig

**Affiliations:** 1 The School of Biological Sciences, the University of Auckland, Auckland 1142, New Zealand; 2 The School of Life Sciences, the University of Lincoln, Lincoln LN6 7DL, UK; 3 Max Planck Institute for Evolutionary Biology, Plön 24306, Germany; 4 Department of Genetics, Evolution, and Environment, University College London, London WC1E 6BT, UK

**Keywords:** Crabtree effect, fermentation, fruit, adaptation, niche, neutral ecology, natural history

## Abstract

Different species are usually thought to have specific adaptations, which allow them to occupy different ecological niches. But recent neutral ecology theory suggests that species diversity can simply be the result of random sampling, due to finite population sizes and limited dispersal. Neutral models predict that species are not necessarily adapted to specific niches, but are functionally equivalent across a range of habitats. Here, we evaluate the ecology of *Saccharomyces cerevisiae*, one of the most important microbial species in human history. The artificial collection, concentration and fermentation of large volumes of fruit for alcohol production produce an environment in which *S. cerevisiae* thrives, and therefore it is assumed that fruit is the ecological niche that *S. cerevisiae* inhabits and has adapted to. We find very little direct evidence that *S. cerevisiae* is adapted to fruit, or indeed to any other specific niche. We propose instead a neutral nomad model for *S. cerevisiae*, which we believe should be used as the starting hypothesis in attempting to unravel the ecology of this important microbe.

## Abstract (precis)

It is assumed that *Saccharomyces cerevisiae* is adapted to inhabit fruits; however, we find very little evidence for adaptation to any niche. Instead, we propose a neutral nomad model for *S. cerevisiae*.

## Graphical abstract

**Graphical Abstract Figure.**
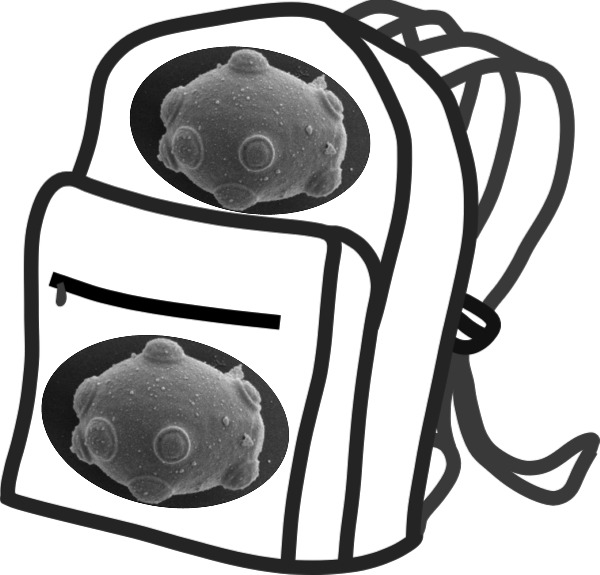
It is assumed that *Saccharomyces cerevisiae* is adapted to inhabit fruits; however, we find very little evidence for adaptation to any niche. Instead, we propose a neutral nomad model for *S. cerevisiae*.

## ADAPTATION AND THE ECOLOGICAL NICHE

The concept of a niche is central to the field of ecology. This concept presumes that there are specific sets of environmental conditions under which different species can thrive, and therefore that there are discrete places and times in which species may be found. Classical ecological theory is in line with this concept and suggests that different species have different sets of ‘functions’, which they acquired under adaptation by natural selection to different ecological niches (Vandermeer 1972). Fundamental constraints restrict the number of functions a single species can have—an organism cannot simultaneously enjoy the benefits of being both big and small for example. Such trade-offs explain biodiversity: any given habitat supports multiple species because no single species can successfully occupy all niches. The metaphor that species have specific functions that allow them to occupy specific niches was developed by direct observation of macroscopic species, and on the whole it satisfactorily explains both biodiversity and why species appear to fit their environments (Vandermeer 1972). This idea has been transferred to the microbial world, where the possibility of huge population sizes and high rates of dispersal should increase the power of natural selection to drive adaptation to specific niches (Hanson *et al.* 2012). This thinking is epitomized by the Baas Becking hypothesis (Baas Becking 1934): ‘everything is everywhere, but the environment selects’. However, direct observations of microbial interactions with their natural environments are often impossible, and it may be that the ecological niche concept is not generally applicable to microbes.

## NEUTRAL THEORY OF ECOLOGY

Recently, other models have been proposed that successfully explain species diversity. Neutral ecology emphasizes the importance of stochastic processes in determining community structure and function (Bell 2001; Hubbell 2005). These models present diversity as the result of random sampling, caused by finite population sizes and limited dispersal. This idea implies that species may not be preferentially adapted to different niches, and that in fact different species might be functionally equivalent across a number of niches. Neutral models of ecology remain controversial and have not yet been widely applied to microbial ecology (Hanson *et al.* 2012), except for examples in which limited dispersal is seen to be a primary determinant of community composition (e.g. Bell 2010; Dumbrell *et al.* 2010; Peay, Garbelotto and Bruns 2010). Here, we reconsider whether the ecological niche metaphor applies to the yeast *Saccharomyces cerevisiae*, one of the best studied laboratory model organisms, but whose ecology and natural history is still largely unknown. We present the idea that *S. cerevisiae* is not adapted to a specific niche, but is instead a nomad that has evolved the general ability to inhabit and persist in many different environments.

## THE IMPORTANCE OF *S. CEREVISIAE*

*Saccharomyces cerevisiae* has been widely used by humans for thousands of years and is arguably one of the most important microbial species in human history (Chambers and Pretorius 2010). It owes this distinction to a single trait: its ability to produce alcohol from sugar. Whilst it is also useful for raising bread, producing fuel and expressing desirable engineered proteins, it was the demand for alcoholic beverages that motivated the scientific study of yeast by Pasteur (1897) and the Carlsberg Research Laboratories (Hansen 1896). Since then *S. cerevisiae* has achieved a second distinction: it is the best understood genetic model organism. *Saccharomyces cerevisiae* was the first eukaryote to have its genome completely sequenced, and its genome is still the best annotated and most tractable to genetic manipulations and analysis (Cherry *et al.* 2011). Huge projects are in the process of determining the biological functions and genetic interactions of every part of the genome (e.g. Kelly, Lamb and Kelly 2001; Boone 2014) on a scale that is unprecedented in any organism. *Saccharomyces cerevisiae* has been key to numerous major breakthroughs in genetics, biochemistry and cell biology (Chambers and Pretorius 2010).

## THE CRABTREE EFFECT

*Saccharomyces cerevisiae* preferentially produces alcohol from sugar by anaerobic fermentation, even when oxygen is available for aerobic respiration. This key trait, known as the Crabtree effect (Pronk, Steensma and Van Dijken 1996), is thought to be an adaptation to high sugar environments. Although fermentation of sugar by *S*. *cerevisiae* is about 10 times less metabolically efficient than aerobic respiration in terms of ATP production, it potentially provides two proposed selective benefits. First, fermentation liberates energy faster and thus enables more rapid growth than aerobic respiration does (Pfeiffer, Schuster and Bonhoeffer 2001). If many individuals compete for a limited shared resource, those that grow more rapidly will win, even if they effectively squander the resource (Pfeiffer, Schuster and Bonhoeffer 2001; MacLean and Gudelj 2006). A useful metaphor for this is ‘the tragedy of the commons’ (Hardin 1968). Secondly, fermentation degrades the environment by producing ethanol, which is not produced by aerobic respiration. In addition, fermentation produces heat and CO_2_ more rapidly than aerobic respiration does, so these may also accumulate. If *S*. *cerevisiae* can tolerate such alcoholic, hot and anoxic environments better than its competitors, then it will enjoy a selective advantage due to the interference effects of its own fermentation, and there is some experimental evidence to support this idea (Goddard 2008). Although often seen as competing hypotheses, these two potential benefits of the Crabtree effect are not mutually exclusive but complementary. Further, having outgrown or interfered with its competitors, *S*. *cerevisiae* can then undergo a ‘diauxic shift’ and switch metabolic gears to use the accumulated ethanol as a substrate for aerobic respiration, recovering some (but not all) of the energy wasted by fermentation (Thomson *et al.* 2005). This reduction of the metabolic cost of initial fermentation is available as a consequence of either or both of the two earlier benefits, but it is usually associated with the second, in the so-called make-accumulate-consume strategy (Piskur *et al.* 2006).

The Crabtree effect is thought to have originated around the time that the ancestor of the *Saccharomyces* clade underwent a whole-genome duplication (Piskur *et al.* 2006). Whilst most duplicated gene copies were subsequently lost, many of the surviving genes play roles in sugar metabolism and may have been maintained because two copies allow increased relative gene expression (Kellis *et al.* 2004). Further, the presence of two copies of a gene allows one to maintain ancestral function, whilst the other is free to diverge and acquire new functions. One such case appears to be the duplicated gene pair *ADH1* and *ADH2* (Thomson *et al.* 2005). *ADH1* reduces acetaldehyde to ethanol during anaerobic respiration. *ADH2*, though, appears to have diverged so that it catalyzes the reverse reaction underpinning the diauxic shift: it reconverts ethanol to acetaldehyde, which can be used to make Acetyl-CoA, which feeds into the citric acid cycle. This neofunctionalization has been proposed as one of the key innovations underlying the Crabtree effect, allowing *S. cerevisiae* both to tolerate ethanol and to recover energy that would otherwise be wasted by fermentation. The fact that the Crabtree effect is thought to have appeared at around the same time as fruiting plants became widespread is cited as evidence that *S*. *cerevisiae* is adapted to a specific niche, fruit. However, evidence is circumstantial: dating the origin of the Crabtree effect in geological time using only genetic data is very error prone, and more recent work suggests that the Crabtree effect may have evolved over a long period of time, and is not just coincidental with the whole-genome duplication (Hagman *et al.* 2013).

## DOES THE SUPERIORITY OF *S. CEREVISIAE* AS A FERMENTER INDICATE THAT IT IS ADAPTED TO FRUIT?

The best evidence that yeasts are adapted to fruit comes from winemaking. When grapes are gathered and crushed, they spontaneously ferment, producing wine (e.g. Goddard 2008). Given the prominence of alcohol in human history (McGovern *et al.* 2004), this basic process must have worked fairly consistently and reliably for a long time, across a wide range of conditions. As *S. cerevisiae* is the primary microbe associated with winemaking, it appears logical to assume that its natural habitat includes grapes and the other fruits that are used to make alcoholic beverages.

However, the natural fruit habitat differs greatly from the artificial fermentation environment created by makers of wine and other traditional alcoholic beverages. Collecting large numbers of individual fruits and crushing them together homogenizes the resource and increases its size, making it equally accessible to all species present. By mixing many individual communities from many individual fruits together into one, the number and diversity of individuals competing increases, and this creates competitive conditions that may favour high rate, rather than high efficiency, of growth (MacLean and Gudelj 2006). Physical containment within a vessel and the decrease in surface area to volume ratio of the sugar resource might enhance the degradation of the environment by preventing ethanol, CO_2_ and heat from dissipating easily as it might from individual fruits in the open, selecting for the interference effects of fermentation. It will also reduce the ability of oxygen to diffuse into the fruit, selecting for fermentation. Winemaking conditions are therefore expected to favour fermentation much more strongly than the conditions on natural fruit, so the success of *S. cerevisiae* in wine does not therefore imply that it is successful on fruit.

The adaptation model predicts that organisms adapted to a niche should be abundant in that niche. But *S. cerevisiae* is in fact vanishingly rare on fruit, even in vineyards where fruiting plants are artificially at very high densities and the associated winemaking would be expected to increase the overall abundance of yeast in the location (Mortimer and Polsinelli 1999; Knight and Goddard 2015). Metagenomic sequencing suggests that *Saccharomyces* sp. comprises less than 1:20 000 of the fungi on ripe grapes in vineyards; instead, Crabtree-negative yeast species dominate (Taylor *et al.* 2014). Other yeast species initially dominate early fermentation of wine, and only after several days of fermentation does *S. cerevisiae* typically become abundant (Goddard 2008; Ciani *et al.* 2010). Indeed, other species may often persist at significant frequency even in the extremely alcoholic conditions at the end of wine fermentation (Goddard 2008; Jolly and Varela 2014). Further, despite artificial conditions that strongly favour fermentation, it is common for spontaneous wine ferments to get ‘stuck’—that is no fermentative microbe dominates and very little ethanol is produced (Bisson and Butzke 2000).

The artificial nature of the conditions that are used to ferment wine and other alcoholic drinks is emphasized by the need for humans to produce alcohol themselves, rather than to collect it from some natural source. Whilst anecdotal reports of animals getting drunk on naturally occurring alcohol are common, well-known examples such as elephants (Morris, Humphreys and Reynolds 2006) and waxwings (Eriksson and Nummi 1983) have been debunked. In addition, although low levels of ethanol can occur in fruits (Eriksson and Nummi 1983) and nectar (Wiens *et al.* 2008), there is no evidence that *Saccharomyces* is the primary microorganism responsible. Thus, although *S. cerevisiae* tends to become the dominant organism when large numbers of fruit are gathered, combined and fermented by winemakers, it does not therefore follow that *S. cerevisiae* is well adapted to fruits under natural conditions. Indeed, given its scarcity on fruits, even in vineyards, it seems reasonable to question whether *S. cerevisiae* is especially adapted to fruit at all.

## IS THE CRABTREE EFFECT A SPANDREL?

In evolutionary biology, a spandrel is a trait that exists as the by-product of the evolution of some other trait, rather than because it was the target of selection. The word refers to architectural spandrels, triangular areas of masonry between structural arches supporting a dome. These alluringly shaped spaces are often highly decorated, and it is tempting to view them as the main feature around which the rest of the building is designed. But this is not so: spandrels exist merely as a necessary by-product of a dome supported by arches. In a classic paper, Gould and Lewontin (1979) used spandrels as an analogy to persuade evolutionary biologists not to view all organisms’ traits as the product of adaptation by natural selection. The Crabtree effect might appear to us to be an important adaptation because of the reverence with which we regard the fermentation of wine, but it is possible that the Crabtree effect did not evolve as an adaptation to ferment natural fruit, or is even as an adaptation at all.

The proposed benefits of the Crabtree effect—rapid growth and interference effects on competitors—have not been quantified experimentally in the natural fruit environment, only in homogenized grape juice in the laboratory (Goddard 2008), or in artificial media (MacLean and Gudelj 2006). Even in fermenting wine, the competitive benefit of ethanol production by *S. cerevisiae* is modest (∼2%), and some yeast species found in spontaneous ferments are not suppressed by ethanol until it exceeds 9%, i.e. close to the end of fermentation (Goddard 2008). Further, fermentation by *S. cerevisiae* occurs even in sugar concentrations 100-fold less than those typically found in fruit (Pfeiffer and Morley 2014). Such low glucose levels would be expected to select for high growth efficiency rather than high growth rate, and the interference effects of fermentation would be negligible at these sugar levels as miniscule levels of ethanol, heat and CO_2_ would be produced. It seems quite possible, therefore, that fermentation has benefits other than high growth rate and interference. For example, perhaps the ethanol serves as an attractant for insect vectors, rather than as an anti-competitor compound (Buser *et al.* 2014). The idea that the Crabtree effect is an adaptation enabling competitive superiority under conditions resembling the artificial winery environment might therefore be a spandrel. Even if the Crabtree effect can be shown to be advantageous under some condition, we cannot easily assess whether natural selection shaped it unless we can assess how often yeast encounters similar conditions in nature.

It is also possible that the Crabtree effect is not a fixed trait in *S. cerevisiae;* however, that the Crabtree effect appears invariant across closely related species make this possibility unlikely. The reason this is a possibility at least is that all *S. cerevisiae* (and for that matter all *Saccharomyces sensu stricto*) have been isolated due to their ability to ferment and gain competitive superiority in sugar-rich environments. This occurred either during spontaneous wine ferments or via enrichment culture isolation procedures, which essentially mimic the winemaking process: typically, environmental samples such as pieces of plant material are placed in a sugary liquid medium and incubated at an elevated temperature. Enrichment cultures are often spiked with ethanol, to favour ethanol-resistant genotypes. Thus, the isolation of *S. cerevisiae* from natural samples itself selects for Crabtree-positive strains, and would likely leave Crabtree-negative strains undetected. Variation in fermentation ability and alcohol tolerance clearly exists even among the current biased sample of *S. cerevisiae* isolates (Stern 2014), and is therefore likely to be greater in nature. Thus, there may well be Crabtree negative, or at least less strongly Crabtree positive, *Saccharomyces* genotypes in nature of which we are unaware.

## WHAT IS *S. CEREVISIAE*'S NICHE?

As we have stated: the adaptation model predicts that organisms should be abundant in niches to which they are adapted. The belief that *S. cerevisiae* is adapted to inhabit fruits derives from its dominance in the spontaneous fermentations of wine. But, as we argue above, the considerable difference between the winery environment and natural fruits, combined with the low abundance of *S. cerevisiae* on fruits, and especially on wine grapes, suggests that this belief may be incorrect. Another feature of fruit is that it is an ephemeral resource. What then does *S. cerevisiae* do when it has exhausted a fruit of nutrients? The simplest explanation is that it disperses to another fruit, perhaps passively or perhaps by an insect vector. Recent work shows that some *S. cerevisiae* release volatile compounds that attract Drosophilid flies, which can vector it from fruit to fruit (Buser *et al.* 2014). But what happens when the fruiting season is over? Diploid yeast cells may undergo meiosis and turn into more resistant haploid spores when starved, so it is possible that once a fruit resource is exhausted, large numbers of yeast cells sporulate and disperse as dormant, resistant spores which might persist for many years, allowing them to survive not only the dispersal process but also the long intervals of time between fruiting seasons. Consistent with this model are the observations that yeast spores, but not vegetatively growing cells, are resistant to *Drosophila* digestion (Reuter, Bell and Greig 2007), and that *S. cerevisiae* has been isolated from *Drosophila* (Buser *et al.* 2014), hibernating wasps (Stefanini *et al.* 2014) and bee hives (Goddard *et al.* 2010)

This raises the possibility that *S. cerevisiae* is adapted to habitats other than fruit, and these may form a refuge when fruit is not available. Finding and characterizing these putative niches is an active field of research. *Saccharomyces cerevisiae* has been isolated from a wide range of environments: in addition to vineyard and winery environments and a range of other human ferments (sake, billi wine, etc.) and baking (Liti *et al.* 2009), *S. cerevisiae* is found, as expected, in fruits and insects, but also in humans as a commensal (Angebault *et al.* 2013) or pathogen (Muller *et al.* 2011), in soil, on various plants (Wang *et al.* 2012) and on oak trees (Sniegowski, Dombrowski and Fingerman 2002; Sampaio and Gonçalves 2008). The belief that oak is another of *S. cerevisiae's* niches comes from a seemingly consistent ability to isolate yeast from oak bark. Unfortunately, it is likely that this survey is biased: many researchers simply want samples of wild yeast to study, and therefore they target environments from which *Saccharomyces* has already been isolated. Since oak trees are typically sympatric with yeast laboratories, and are easy for microbiologists to identify, it is not surprising that they are a favoured source of wild yeast samples.

The enrichment culture method that is nearly always used to isolate yeast also likely causes severe biases, not only in favour of Crabtree-positive yeast, as discussed above, but also in obscuring the true distribution and range of *S. cerevisiae*. If a sample does not yield *S. cerevisiae* by enrichment culture, it does not mean that it was not present or viable, but only that it did not grow sufficiently to outcompete the other microbes in the sample. Nor is it possible to tell whether any *S. cerevisiae* strains that are recovered originated from dormant spores in the sample, or from an actively growing population. The observed homozygosity of soil and wild oak-tree-associated *Saccharomyces* might therefore be an artefact of the isolation process, if isolates are derived from rare single spores that autodiploidize in the enrichment culture by mating-type switching (Goddard *et al.* 2010). Consistent with this explanation, mitotic diploids isolated directly from wine ferments are typically much more heterozygous than those from oak bark and soil (Goddard *et al.* 2010; Knight and Goddard 2015).

Simple enrichment culture does not give any indication of the abundance of *S. cerevisiae* in a primary sample. By determining the sensitivity of enrichment culture to detect single yeast cells spiked into oak bark samples, and making appropriate dilutions of oak bark samples, we have estimated the average density of *S. paradoxus*, *S. cerevisiae*'s sister species, to be just two cells per square centimetre of oak bark; consistent with this extremely low density, we detected no *Saccharomyces* sequences at all among 40 000 fungal sequences extracted from oak bark (Kowallik, Miller and Greig 2015). We are unaware of such estimates for *S. cerevisiae*. Further, recent work shows that some *S. cerevisiae* isolates grow poorly in ‘oak bark extract’ in the laboratory (Giraldo-Perez and Goddard 2013). Together, evidence suggests that oak bark is not a niche that *S. cerevisiae* is especially abundant in or well adapted to.

## GENETIC VARIATION WITHIN *S. CEREVISIAE*

The global population structure of *S. cerevisiae* shows evidence for clades associated with specific environments, consistent with adaptation of certain genotypes to certain environments (Liti *et al.* 2009). But there is also an equal weight of evidence for isolation by distance, consistent with neutral divergence due to limited dispersal and subsequent lack of gene flow between geographically remote populations (Liti *et al.* 2009; Knight and Goddard 2015). Limited sampling of such wild populations by humans making artificial fermentations could result in the association between genotype and environment appearing by chance, rather than by adaptation. The prime case is for the population inhabiting Europe. Humans inadvertently amplified this subpopulation with their winemaking, and provided an increased density of opportunities for *S. cerevisiae* at some point ∼9000 years ago when we began to deliberately grow fruit and make wine in one place (Legras *et al.* 2007). This Wine/European lineage is now no longer geographically constrained because humans have transported it around the globe along with viticulture and wine making (Legras *et al.* 2007; Liti *et al.* 2009; Goddard *et al.* 2010; Gayevskiy and Goddard in preparation). Whilst this *S. cerevisiae* lineage now has one apparent adaptation to agricultural interventions, resistance to copper and sulphur that are used as antimicrobials in vineyards (Aa *et al.* 2006), there is no other compelling evidence that the founders of this lineage were differentially adapted or better at fermenting than individuals from other lineages. A possible way to determine whether wild populations are adapted to specific habitats is to use a form of reciprocal transplant experiment. For example, a set of strains isolated from different habitats could be tested in direct competition assays in conditions simulating the different habitats. If strains tend to have high relative fitness in the environment they were isolated from but low relative fitness elsewhere, it would indicate that they were indeed adapted to specific conditions.

## THE NOMAD MODEL

The ripe speculation in these last paragraphs indicates our lack of data concerning *S. cerevisiae*'s natural history. We have little direct evidence concerning the niche or niches that *S. cerevisiae* might be abundant in or adapted to. We know little about its distribution, or the form it takes in different habitats (dormant spores or vegetative cells); nor do we know how the asexual, sexual and dormant phases of its life cycle fit into its life history. *Saccharomyces cerevisiae* is known to be abundant only in the ferments of artificially gathered fruit, but it appears to be sparsely distributed everywhere else that has been surveyed for, and particularly sparse on fruits and oak tree bark, the most commonly claimed niches for this species.

This leads us to propose an alternative neutral model: that *S. cerevisiae* is not adapted to a specific niche, but is a nomad, able to survive as a generalist at low abundance in a wide range of environments. This is consistent with the observation that *S. cerevisiae* can be found in a diverse range of habitats. The low abundance of this species is also consistent with this it being a generalist, capable of doing lots of things, but none of them especially well. *Saccharomyces cerevisiae* has a rich metabolism that enables it to survive or grow in a wide range of environments eclipsing those found in either fruits or bark, with varying nutrient availabilities—with both low and high carbon and nitrogen concentrations (Wenger *et al.* 2011; Gray *et al.* 2012); pH—from strongly acidic ∼pH 3 (Goddard 2008) to alkaline ∼pH8 (Serrano *et al.* 2006); osmolarity—from survival in water through to NaCl concentrations of at least 1.3 M (Petrovska, Winkelhausen and Kuzmanova 1999); and temperatures ranging from close to 0 to around 45°C (Sweeney *et al.* 2004; Salvado *et al.* 2011). These observations suggest that the fundamental niche, the set of conditions where *S. cerevisiae* may survive, is very broad, and this is in line with it being a generalist. Comprehensive fitness measurements across a range of conditions (including interactions with other species), for a wide range of genotypes, may indicate the realized niche for *S. cerevisiae*: the range of conditions actually used by this species. Such a task is not trivial, however. Lastly, there is genomic evidence that *S. cerevisiae* is a generalist, not a specialist: the genome is complex containing 6000 genes, of which only 20% are necessary for growth in simple laboratory medium (Giaever *et al.* 2002).

*Saccharomyces cerevisiae*'s diverse metabolic tolerances, range of habitats of isolation and low densities are therefore consistent with a nomad model, but they are not inconsistent with it being adapted to some other, as yet undetermined niche or niches. The main utility of the Nomad Model, then, is as a neutral scientific starting point, which can serve as a null hypothesis for evaluating adaptive explanations for *S. cerevisiae* evolution. Rather than assuming that *S. cerevisiae* is adapted to a niche, and then doggedly search for data that back up this prejudice, we should start from the position of neutrality.

Future methods that could be used to test proposed niche models against the neutral Nomad Model include experimental evolution in a candidate niche to test whether genes are maintained by selection in a given environment such as grape juice, or whether superfluous pathways are lost, indicating that the tested environment is not a niche that *S. cerevisiae* is adapted to. Unbiased surveys—that sample niches systematically, regardless of where *S. cerevisiae* has previously been discovered—will provide data as to the incidence of this species in various habitats. The increasing torrent of environmental sequence data will also provide opportunities for realistic estimates for *S. cerevisiae*'s abundances. The Nomad Model can be rejected if comprehensive, systematic and unbiased surveys reveal that *S. cerevisiae* inhabits and is competitively robust in specific natural habitats and not others. Predictable changes in abundance indicating seasonal growth cycles would also be valuable evidence supporting adaptation. Such data could explain how the life cycle of *S. cerevisiae* fits in with the changes in its natural environment.

## CONCLUSION

It is tempting to apply the niche adaptation concept to *S. cerevisiae*, and to microbes in general, as this concept is familiar to us by observation of large organisms. However, we must be cautious not to assume adaptation to specific niches, but demand evidence for such adaptations. The nomad model serves as a neutral model for *S. cerevisiae*, and is the counterpoint to the assertion that the species is adapted to one or more specific niches. We believe that it should be used as the null hypothesis for research attempting to unravel the ecology of this important model microbe.
